# Disruption of Otoferlin Alters the Mode of Exocytosis at the Mouse Inner Hair Cell Ribbon Synapse

**DOI:** 10.3389/fnmol.2018.00492

**Published:** 2019-01-09

**Authors:** Hideki Takago, Tomoko Oshima-Takago, Tobias Moser

**Affiliations:** ^1^Institute for Auditory Neuroscience and InnerEarLab, University Medical Center Göttingen, Göttingen, Germany; ^2^Department of Rehabilitation for Sensory Functions, Research Institute, National Rehabilitation Center for Persons with Disabilities, Saitama, Japan; ^3^Collaborative Research Center 889 Cellular Mechanisms of Sensory Processing, Göttingen, Germany; ^4^Göttingen Graduate School for Neurosciences and Molecular Biosciences, University of Göttingen, Göttingen, Germany; ^5^Auditory Neuroscience Group, Max Planck Institute for Experimental Medicine, Göttingen, Germany; ^6^Synaptic Nanophysiology Group, Max Planck Institute for Biophysical Chemistry, Göttingen, Germany

**Keywords:** auditory, cochlea, hair cell, spiral ganglion neuron, ribbon synapse, otoferlin, calcium, EPSC

## Abstract

Sound encoding relies on Ca^2+^-mediated exocytosis at the ribbon synapse between cochlear inner hair cells (IHCs) and type I spiral ganglion neurons (SGNs). Otoferlin, a multi-C_2_ domain protein, is proposed to regulate Ca^2+^-triggered exocytosis at this synapse, but the precise mechanisms of otoferlin function remain to be elucidated. Here, performing whole-cell voltage-clamp recordings of excitatory postsynaptic currents (EPSCs) from SGNs in otoferlin mutant mice, we investigated the impact of *Otof* disruption at individual synapses with single release event resolution. *Otof* deletion decreased the spontaneous release rate and abolished the stimulus-secretion coupling. This was evident from failure of potassium-induced IHC depolarization to stimulate release and supports the proposed role of otoferlin in Ca^2+^ sensing for fusion. A missense mutation in the *Otof* gene (pachanga), in which otoferlin level at the IHC plasma membrane was lowered without changing its Ca^2+^ binding, also reduced the spontaneous release rate but spared the stimulus-secretion coupling. The slowed stimulated release rate supports the hypothesis that a sufficient abundance of otoferlin at the plasma membrane is crucial for the vesicle supply. Large-sized monophasic EPSCs remained present upon *Otof* deletion despite the drastic reduction of the rate of exocytosis. However, EPSC amplitude, on average, was modestly decreased. Moreover, a reduced contribution of multiphasic EPSC was observed in both *Otof* mutants. We argue that the presence of large monophasic EPSCs despite the exocytic defect upon *Otof* deletion supports the uniquantal hypothesis of transmitter release at the IHC ribbon synapse. Based upon the reduced contribution of multiphasic EPSC, we propose a role of otoferlin in regulating the mode of exocytosis in IHCs.

## Introduction

Ca^2+^ influx and subsequent neurotransmitter release at inner hair cell (IHC) active zones govern sound encoding at the first auditory synapse. Excitatory postsynaptic currents (EPSCs) recorded from type I spiral ganglion neurons (SGNs) show remarkable variability in amplitude and shape. This EPSC heterogeneity led to the hypothesis of synchronized multiquantal release (MQR) at hair cell synapses (Glowatzki and Fuchs, 2002; Keen and Hudspeth, 2006; Goutman and Glowatzki, 2007; Neef et al., 2007; Li et al., 2009; Grant et al., 2010; Graydon et al., 2011; Schnee et al., 2013). MQR would cause monophasic (temporally compact) EPSCs when the exocytosis of vesicles comprising a MQR event is highly synchronized or multiphasic (temporally non-compact) EPSCs when synchronization of MQR is poor. However, whereas Ca^2+^ influx by presynaptic depolarization increases the EPSC amplitude at the frog hair cell synapse (Li et al., 2009; Graydon et al., 2011), neither changes in voltage-gated Ca^2+^ influx (Glowatzki and Fuchs, 2002; Grant et al., 2010) nor those in presynaptic Ca^2+^ buffering (Goutman and Glowatzki, 2007) affects the EPSC amplitude distribution at the rat IHC ribbon synapse. Strikingly, even when the presynaptic Ca^2+^ influx was abolished in mouse IHCs, EPSC size remained heterogeneous and the charge distribution of mono- and multiphasic EPSCs were unchanged (Chapochnikov et al., 2014). This, together with other experimental observations and modeling, led to the proposal that uniquantal release (UQR) is a candidate mechanism for exocytosis at the mammalian IHC ribbon synapse, whereby a combination of large clusters of postsynaptic AMPA receptors and presynaptic fusion pore dynamics would generate large and variably shaped EPSCs (Chapochnikov et al., 2014; for review, see Takago and Oshima-Takago, 2018). The UQR hypothesis of IHC exocytosis has received further support by recent studies manipulating presynaptic IHC Ca^2+^ influx (Huang and Moser, 2018) or directly measuring membrane fusion steps via cell-attached membrane capacitance recordings from IHCs (Grabner and Moser, 2018).

Disruption of *OTOF*, coding for otoferlin, was identified to cause hereditary deafness DFNB9 (Yasunaga et al., 1999), while missense mutations of *OTOF* can lead to less profound hearing impairment (Varga et al., 2003; Marlin et al., 2010; Vogl et al., 2016; for review, see Pangršič et al., 2012; Moser and Starr, 2016). As a multi-C_2_ domain protein, otoferlin, in analogy to synaptotagmins, was initially proposed to serve as a Ca^2+^ sensor for fusion at the IHC ribbon synapse based on a functional analysis of a mouse line with a null mutation in otoferlin (*Otof^-/-^*, Roux et al., 2006) and biochemical studies (Johnson and Chapman, 2010). Moreover, a role of otoferlin in efficient vesicle priming is proposed based upon an analysis of a mouse line called pachanga that carries a missense mutation in otoferlin C_2_F domain (*Otof^D1767G/D1767G^* or *Otof^Pga/Pga^*, Pangršič et al., 2010). Whereas such a mutation does not affect Ca^2+^ binding, *Otof^Pga/Pga^* IHCs show reduced membrane-bound otoferlin level but unaltered fusion, thus suggesting that the hearing impairment arises from strongly reduced vesicle replenishment (Pangršič et al., 2010). This hypothesis is further supported by analyses of mouse lines carrying a missense mutation in otoferlin C_2_C domain that again lowers membrane-bound otoferlin level but unaltered fusion (*Ofof^I515T/I515T^*, Strenzke et al., 2016) and a null mutation in transmembrane recognition complex40 receptor tryptophan-rich basic protein that is essential for the insertion of otoferlin into the endoplasmic reticulum in IHCs (Vogl et al., 2016). Thus, the abundance of otoferlin in IHCs is critical for maintaining the vesicle resupply to the ribbon-type active zone. Otoferlin’s function in vesicle replenishment appears to involve the regulation of short filamentous tethering formation between synaptic vesicles and the active zone membrane (Vogl et al., 2015) and the facilitation of clearance of vesicular release sites from previously exocytosed membranes (exocytosis-endocytosis coupling) via an interaction with the endocytic adaptor protein 2 (Duncker et al., 2013; Jung et al., 2015). On the other hand, a recent study utilizing mice with double missense mutations in otoferlin C_2_C domain that affect Ca^2+^ sensing (*Otof^D515A,D517A/D515A,D517A^*, Michalski et al., 2017) has reported that fast and sustained components of release are reduced despite unaltered otoferlin level, probably due to dysfunctional Ca^2+^ binding of otoferlin.

In order to further examine the otoferlin’s function we performed postsynaptic voltage-clamp recordings from afferent boutons of type I SGNs in wild-type (*Otof ^+/+^*), *Otof^Pga/Pga^* and *Otof^-/-^* mice and investigated exocytosis at the levels of single synapses and single release events. Analyzing the spontaneous and stimulated release rates as well as the amplitude and shape of EPSCs, we find evidence for roles of otoferlin in Ca^2+^-dependent fusion and replenishment of vesicles. Moreover, we propose an additional role of otoferlin in regulating the exocytic mode of IHCs to facilitate multiphasic EPSCs, potentially by controlling the vesicle fusion pore during uniquantal release at the IHC ribbon synapse.

## Materials and Methods

### Ethics Statement

All experiments complied with national animal care guidelines in Germany and Japan and were approved by the University of Göttingen board for animal welfare together with the animal welfare office of the state of Lower Saxony (Germany) as well as National Rehabilitation Center for Persons with Disabilities animal experimentation committee (Japan).

### Animals and Preparations

Postnatal day (P) 8–11 mice of either sex were used. Generation and general description of *Otof^Pga/Pga^* (Schwander et al., 2007) and *Otof^-/-^* (Reisinger et al., 2011) mice were previously provided. As wild-type controls, C57BL6 mice (*Otof ^+/+^*), which were not littermates of *Otof^Pga/Pga^* or *Otof^-/-^* mice, were employed. In total, 33 mice (13 *Otof ^+/+^*, 7 *Otof^Pga/Pga^* and 13 *Otof^-/-^* mice, respectively) were used in the present study. After decapitation under deep carbon dioxide inhalation anesthesia, the apical coils of organ of Corti were harvested out of the cochlea.

### Electrophysiology

Whole-cell voltage-clamp recordings from postsynaptic boutons of mouse type I spiral ganglion neurons in apical coils of freshly dissected organ of Corti were performed as previously described for rats (Glowatzki and Fuchs, 2002; Rutherford et al., 2012) and for mice (Pangršič et al., 2010; Jing et al., 2013; Chapochnikov et al., 2014). The recording pipette resistance was 8–15 MΩ after pressure polishing (Goodman and Lockery, 2000). The intracellular solution contained (in mM): 150 CsCl (or 150 KCl in some recordings), 3.5 MgCl_2_, 0.1 CaCl_2_, 5 EGTA, 5 HEPES, and 2.5 Na_2_ATP, pH 7.2. The extracellular solution (artificial perilymph, aPL) for both dissection and recording contained (in mM): 5.8 KCl, 155 NaCl, 0.9 MgCl_2_, 1.3 CaCl_2_, 0.7 NaH_2_PO_4_, 5.6 D-glucose, and 10 HEPES, pH 7.4. In the high K^+^ extracellular solution to depolarize presynaptic IHCs, 40 out of 155 mM NaCl were replaced with equimolar KCl. In most recordings, tetrodotoxin (1–2 μM) was added to block voltage-gated Na^+^ channels. Currents were low-pass filtered at 5–10 kHz and sampled at 20–50 kHz. EPSCs were recorded at a holding potential of -90 mV (∼4 mV liquid junction potential not corrected) at room temperature (21–24°C).

### Chemicals and Equipment

All chemicals were purchased from Sigma-Aldrich (St. Louis, MO, United States) except for tetrodotoxin (Tocris Bioscience, Bristol, United Kingdom or Wako Pure Chemical Industries, Ltd., Osaka, Japan). The EPC-10 amplifier controlled by Patchmaster software (HEKA Elektronik, Lambrecht, Germany) as well as an upright microscope with differential interference contrast optics (Axioskop FS2, Carl Zeiss, Oberkochen, Germany or BX51WI, Olympus, Tokyo, Japan) was used.

### Data Analysis and Statistics

For detection and analysis of EPSCs, MiniAnalysis software (Synaptosoft, Decatur, GA, United States) was used with a detection threshold set at 3–5 times greater than the root mean square (rms) of the baseline noise. To classify EPSCs into mono- or multiphasic, the methods introduced by Grant et al. (2010) as well as Chapochnikov et al. (2014) were employed. For plotting, IGOR Pro (Wavemetrics, Lake Oswego, OR, United States), Sigmaplot (Systat Software Inc., San Jose, CA, United States) were used. The passive membrane properties such as series resistance (R_s,_ 40 ± 2 MΩ for 13 *Otof ^+/+^* SGNs, 41 ± 3 MΩ for 7 *Otof^Pga/Pga^* SGNs, 40 ± 2 MΩ for 13 *Otof^-/-^* SGNs), membrane capacitance (C_m_, 1.9 ± 0.2 pF for *Otof ^+/+^*, 1.9 ± 0.1 pF for *Otof^Pga/Pga^*, 1.8 ± 0.2 pF for *Otof^-/-^*) and membrane input resistance (R_m,_ 498 ± 141 MΩ for *Otof ^+/+^*, 831 ± 225 MΩ for *Otof^Pga/Pga^*, 651 ± 106 MΩ for *Otof^-/-^*) were calculated as previously described (Chapochnikov et al., 2014). Recordings with R_s_ > 50 MΩ or less than 20 EPSCs were excluded from the EPSC amplitude/charge or kinetics analysis, but included in the EPSC frequency analysis (except for recordings with less than 5 EPSCs) not to ignore very low-frequency auditory nerve fibers. Data is shown as mean ± SEM. Statistical significance was evaluated by Student’s *t*-test or one way ANOVA followed by *post-hoc* Tukey’s test.

## Results

### Missense Mutation of Otoferlin Decreases the Rate of Spontaneous Release, Loss of Otoferlin Abolishes Stimulus-Secretion Coupling in IHCs

**FIGURE 1:**
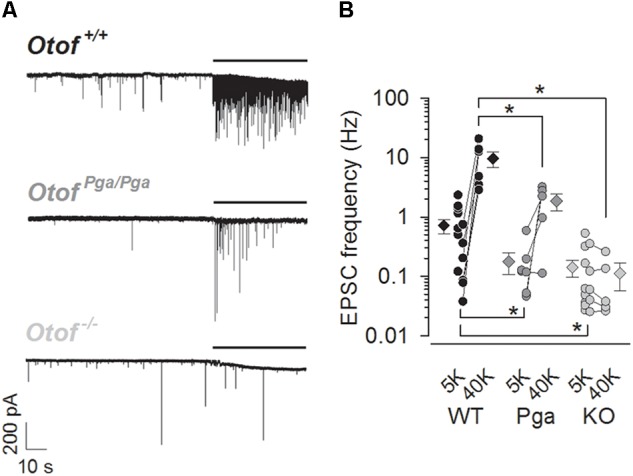
Otoferlin regulates spontaneous and high K^+^-stimulated release at the mouse IHC ribbon synapse. **(A)** Sample traces of EPSCs recorded from exemplar boutons of type I SGNs from a P9 *Otof ^+/+^* (top), a P8 *Otof^Pga/Pga^* (middle) and a P9 *Otof ^-/-^* (bottom) mouse. Each recording started in the extracellular solution containing 5.8 mM KCl, and 40 mM KCl was bath-applied during the time indicated by horizontal bars to depolarize presynaptic IHCs. **(B)** Summary of EPSC frequency in 5.8 mM (5K, spontaneous rate) and 40 mM (40 K, stimulated rate) [K^+^]_e_. Circles and diamonds indicate individual and mean ± SEM values for *Otof ^+/+^* (WT, black), *Otof^Pga/Pga^* (Pga, dark gray) and *Otof ^-/-^* (KO, light gray) SGNs. Asterisks at the bottom (spontaneous rate) and top (stimulated rate) show significant differences (^∗^*p* < 0.05). Note that the vertical axes for this and the next panels are shown on logarithmic scale. In some recordings, where 40 mM KCl was not applied, data points for 5.8 mM [K^+^]_e_ alone were plotted.

EPSCs were recorded from afferent boutons of type I SGNs in P8–11 *Otof ^+/+^*, *Otof^Pga/Pga^* (missense mutation with reduced abundance of otoferlin) and *Otof^-/-^* mice. At rest (1.3 mM Ca^2+^ and 5.8 mM K^+^ in aPL), SGNs of all three genotypes steadily exhibited spontaneous EPSCs (Figure 1A), but their rate was reduced in SGNs of both mutants. The frequencies of spontaneous EPSCs were 0.71 ± 0.19 Hz for *Otof ^+/+^* (*n* = 13, Figure 1B), 0.18 ± 0.07 Hz for *Otof^Pga/Pga^* (*n* = 7, *p* = 0.048 compared to *Otof ^+/+^*) and 0.14 ± 0.04 Hz for *Otof^-/-^* SGNs (*n* = 13, *p* = 0.010 and 0.985 compared to *Otof ^+/+^* and *Otof^Pga/Pga^*, respectively). Hence, unlike disruption of synaptotagmin I or II at the central synapses, which is thought to unclamp spontaneous release (Nishiki and Augustine, 2004; Pang et al., 2006; Xu et al., 2009; Kochubey and Schneggenburger, 2011; Lee et al., 2013; Liu et al., 2014), *Otof* deletion did not increase the spontaneous EPSC frequency. This argues against a clamping function of otoferlin for spontaneous release and highlights the importance of otoferlin in vesicle replenishment, since the spontaneous rate was reduced to a similar extent in *Otof^-/-^* and *Otof^Pga/Pga^* mice, which in a previous study showed intact vesicle fusion but impaired replenishment in IHCs (Pangršič et al., 2010).

Next, we examined the stimulus-secretion coupling in IHCs. High K^+^ solution (40 mM in aPL) was bath-applied onto IHCs to increase the open probability of their Ca_V_1.3 L-type Ca^2+^ channels. The frequency of EPSCs in *Otof ^+/+^* SGNs was increased from 0.25 ± 0.11 (5.8 mM, control) to 9.67 ± 2.90 Hz (first 10 s during high K^+^ stimulation, *n* = 6, *p* = 0.021, Student’s paired *t*-test, Figures 1A,B). Here, the spontaneous EPSC frequency for this control (5.8 mM K^+^) was undervalued due to our preferential application of high K^+^-containing aPL onto low-frequency synapses, since remaining more active synapses (1.10 ± 0.28 Hz, *n* = 7) provided a sufficient number of EPSCs for the analyses of EPSC size and shape even in the normal K^+^-containing aPL. In *Otof^Pga/Pga^* SGNs, high K^+^ stimulation increased the frequency of EPSCs in 4 out of 5 *Otof^Pga/Pga^* SGNs from 0.20 ± 0.10 (control) to 1.86 ± 0.57 Hz (high K^+^, *n* = 5, *p* = 0.047), indicating that the missense *Otof* mutation in *Otof^Pga/Pga^* mice spares stimulus-secretion coupling in IHCs. The reduced rate of stimulated release in *Otof^Pga/Pga^* IHCs (*p* = 0.032 compared to *Otof ^+/+^*) is compatible with impaired vesicle replenishment (Pangršič et al., 2010). In contrast, the high K^+^ stimulation failed to increase the EPSC frequency in *Otof^-/-^* SGNs (control: 0.12 ± 0.06 Hz vs. high K^+^: 0.10 ± 0.05 Hz, *n* = 5, *p* = 0.302), indicating that *Otof* deletion abolishes stimulus-secretion coupling in IHCs. The difference in stimulated release rate between *Otof ^+/+^* and *Otof^-/-^* SGNs again showed significance (*p* = 0.010).

### Otoferlin Disruption Decreases EPSC Amplitude and the Fraction of Multiphasic EPSCs

We studied the effects of *Otof* disruption on the size and shape of EPSCs. As reported in previous studies using the organ of Corti of rats (Glowatzki and Fuchs, 2002; Grant et al., 2010), high K^+^ stimulation of mouse *Otof ^+/+^* organ of Corti increased the frequency of EPSCs without affecting the EPSC size (monophasic EPSC amplitude: 141 ± 31 pA for control vs. 157 ± 24 pA for high K^+^, multiphasic EPSC amplitude: 101 ± 13 pA for control vs. 109 ± 16 pA for high K^+^) or the fraction of multiphasic EPSCs (41.9 ± 3.8 % for control vs. 41.1 ± 1.7 % for high K^+^). Moreover, EPSC size and kinetics distributions were unaltered by high K^+^ stimulation in each phenotype SGNs (Supplementary Figures S1, S2). Therefore, EPSCs recorded in control and high K^+^ were pooled for subsequent analyses in this study.

**FIGURE 2:**
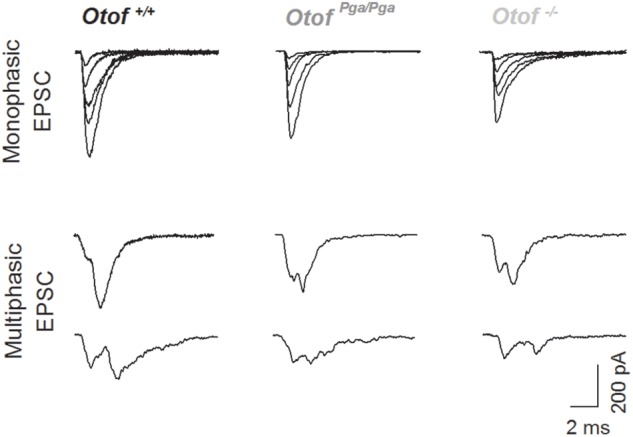
Deletion of otoferlin does not abolish large EPSCs. Sample traces of monophasic (top row) and multiphasic (bottom rows) EPSCs recorded from *Otof ^+/+^* (left), *Otof ^Pga/Pga^* (middle) and *Otof ^-/-^* (right) SGNs.

**FIGURE 3:**
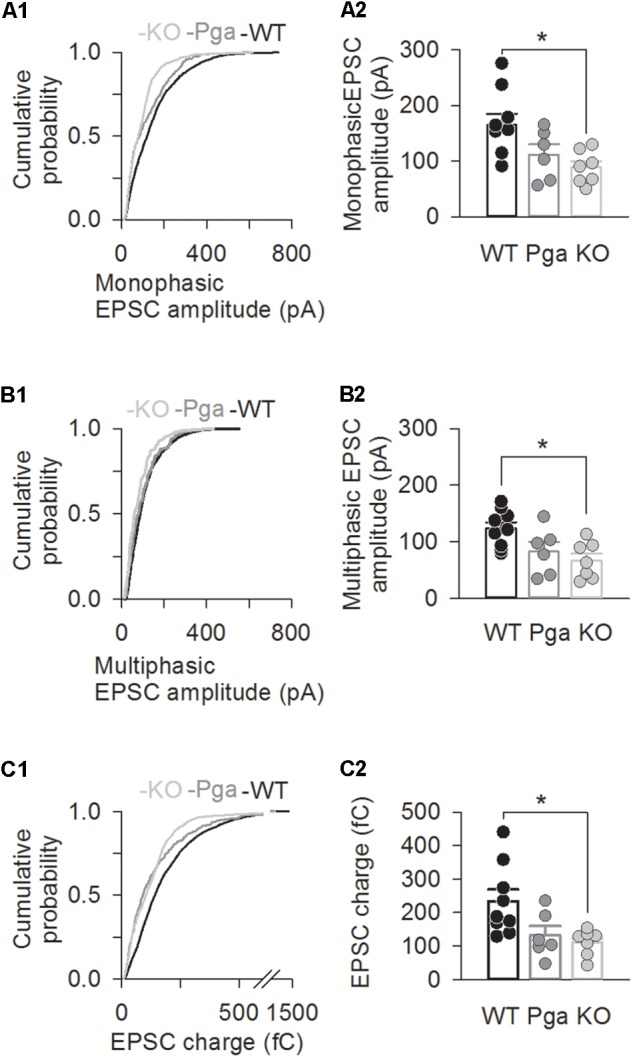
Otoferlin deletion decreases EPSC amplitude and charge. **(A–C)** EPSC size for *Otof ^+/+^* (black), *Otof ^Pga/Pga^* (dark gray) and *Otof ^-/-^* (light gray) SGNs. Significant differences between *Otof ^+/+^* and *Otof^-/-^* SGNs (^∗^*p* < 0.05). Cumulative histograms of monophasic EPSC amplitude (**A1)**, multiphasic EPSC amplitude **(B1)** and mono- and multiphasic (mixed) EPSC charge **(C1)** as well as individual (circles) and mean ± SEM (bars) values of monophasic EPSC amplitude **(A2)**, multiphasic EPSC amplitude **(B2)** and mono- and multiphasic EPSC charge **(C2)** are shown. Note that a break line is inserted in the horizontal axis of panel C2 to clearly demonstrate the differences among groups.

**FIGURE 4:**
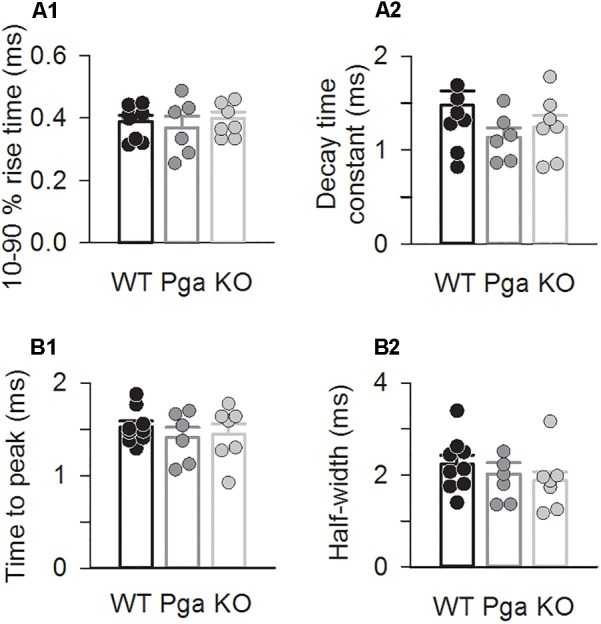
Otoferlin disruption does not change EPSC kinetics **(A,B)** EPSC kinetics for *Otof ^+/+^* (black), *Otof ^Pga/Pga^* (dark gray) and *Otof ^-/-^* (light gray) SGNs. Individual (circles) and mean ± SEM (bars) values of 10–90 % rise time **(A1)** and decay time constant **(A2)** of monophasic EPSCs as well as time to peak **(B1)** and half-width **(B2)** of multiphasic EPSCs. No significant differences.

Surprisingly, despite the reduction in release rate and the lack of stimulus-secretion coupling, *Otof^-/-^* synapses showed variable EPSC amplitudes from 11 up to several hundred pA (Figure 2). As shown in Figure 3 and Table 1, the average amplitudes of mono- and multiphasic EPSCs as well as the average charge transfer of mixed EPSCs were reduced in *Otof^-/-^* SGNs (*p* = 0.013 for monophasic EPSC amplitude, *p* = 0.015 for multiphasic EPSC amplitude, *p* = 0.011 for EPSC charge). There was a trend to smaller EPSC amplitudes also for *Otof^Pga/Pga^* SGNs, which, however, did not reach significance. EPSC kinetics (i.e., rise time and decay time for monophasic as well as time to peak and half width for multiphasic), on the other hand, was not different among those three groups (Figures 2, 4).

**Table 1:** EPSC size in SGN afferent boutons of wild-type and Otof mutant mice.

	Monophasic EPSC	Multiphasic EPSC	EPSC charge
	amplitude (pA)	amplitude (pA)	transfer (fC)
*Otof ^+/+^*	166 ± 20	123 ± 11	234 ± 35
(*n* = 9)	(1473 EPSCs)	(1049 EPSCs)	(2522 EPSCs)
*Otof^Pga/Pga^*	112 ± 18	83 ± 17	132 ± 28
(*n* = 6)	(301 EPSCs)	(100 EPSCs)	(401 EPSCs)
*Otof^-/-^*	89 ± 11	67 ± 12	103 ± 13
(*n* = 7)	(479 EPSCs)	(101 EPSCs)	(580 EPSCs)


**FIGURE 5:**
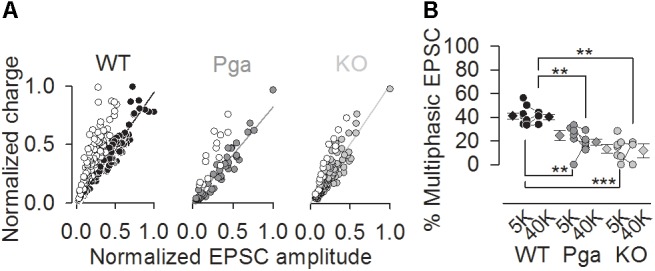
Otoferlin disruption decreases the fraction of multiphasic EPSCs **(A,B)** Exocytic mode of IHC exocytosis for *Otof ^+/+^* (black), *Otof ^Pga/Pga^* (dark gray) and *Otof ^-/-^* (light gray) SGNs. **(A)** Scatter plot of amplitude versus charge of monophasic (filled circles) and multiphasic (open circles) EPSCs derived from exemplar *Otof ^+/+^*, *Otof^Pga/Pga^* and *Otof ^-/-^* SGNs. Note that the *Otof^Pga/Pga^* and *Otof ^-/-^* EPSCs cluster predominantly around the regression lines for monophasic EPSCs, which is indicative of less multiphasic EPSCs in *Otof* mutants than in *Otof ^+/+^*. **(B)** Summary of the EPSC mode in 5.8 mM (5K, spontaneous rate) and 40 mM (40K, stimulated rate) [K^+^]_e_. Circles and diamonds indicate individual and mean ± SEM values of the percentage of multiphasic EPSCs for *Otof ^+/+^* (WT, black), *Otof^Pga/Pga^* (Pga, dark gray) and *Otof ^-/-^* (KO, light gray) SGNs. Asterisks at the bottom (spontaneous rate) and top (stimulated rate) show significant differences (^∗∗^*p* < 0.01 and ^∗∗∗^*p* < 0.001).

Notably, the scatter plot of EPSC amplitude versus charge shows the predominance of monophasic EPSCs clustering around the unity line in SGNs of both *Otof* mutants, while a substantial group of multiphasic EPSCs with smaller amplitude but similar charge exists in *Otof ^+/+^* SGNs (Figure 5A). The fraction of multiphasic EPSC in normal K^+^-containing aPL was significantly reduced in SGNs of both mutants (41.3 ± 2.6% for *Otof ^+/+^*, 24.0 ± 5.2% for *Otof^Pga/Pga^* and 13.2 ± 3.5% for *Otof^-/-^*, *p* < 0.01 between *Otof ^+/+^* and *Otof^Pga/Pga^*, *p* < 0.001 between *Otof ^+/+^* and *Otof^-/-^*, *p* = 0.145 between *Otof^Pga/Pga^* and *Otof^-/-^*, Figure 5B). Also, the fraction of multiphasic EPSC in high K^+^-containing aPL was also significantly reduced in both mutant**s** (40.3 ± 2.3% for *Otof ^+/+^*, 18.7 ± 2.9% for *Otof^Pga/Pga^* and 11.8 ± 5.9% for *Otof^-/-^*, *p* < 0.01 between *Otof ^+/+^* and *Otof^Pga/Pga^*, *p* < 0.01 between *Otof ^+/+^* and *Otof^-/-^*, *p* = 0.408 between *Otof^Pga/Pga^* and *Otof^-/-^*). Thus, monophasic EPSCs dominate transmission in the absence of otoferlin, suggesting that otoferlin regulates the mode of spontaneous and stimulated release at the first auditory synapse.

## Discussion

### Roles of Otoferlin for Stimulus-Secretion Coupling and Vesicle Replenishment

In the present study, we recorded EPSCs from the postsynaptic afferent boutons of type I SGNs in wild-type and otoferlin mutant mice, and tested the function of otoferlin in exocytosis at the IHC ribbon synapse. We found a complete disruption of stimulus-secretion coupling in *Otof^-/-^* SGNs. As Ca^2+^ influx and vesicle availability on a morphological level are maintained at the AZs of *Otof^-/-^* IHCs (Roux et al., 2006), we argue that Ca^2+^ no longer efficiently drives fusion in the absence of otoferlin. This supports the role of otoferlin as a Ca^2+^ sensor of fusion (Roux et al., 2006; Johnson and Chapman, 2010; Michalski et al., 2017). But how is otoferlin-independent spontaneous release regulated? Possible mechanisms include (1) an additional high affinity Ca^2+^ sensor whose capacity is saturated at resting [Ca^2+^]_i_ and (2) a Ca^2+^-independent release process. For (1), the activation range of the remaining Ca^2+^ sensor should be below the resting [Ca^2+^]_i_ of a few tens of nanomolar (Beutner and Moser, 2001). However, to date, there has been no report about such a molecule that meets this requirement. The closest candidate Ca^2+^ sensor for fusion is the C_2_ domain protein Doc2b that promotes membrane fusion at submicromolar Ca^2+^ concentrations (but higher than 100 nM) (Groffen et al., 2010).

Interestingly, the absence of otoferlin did not increase the spontaneous EPSC frequency, which is in contrast to the effect of ablation of synaptotagmin I (Xu et al., 2009) or II (Pang et al., 2006) in the mouse central neurons, further highlighting differences among otoferlin and synaptotagmin I (Reisinger et al., 2011). We note here that the “spontaneous” release from resting IHCs likely includes Ca^2+^-evoked release that is triggered by rare openings of Ca_V_1.3 channels. We consider it likely that the reduction of spontaneous release reflects a combination of disrupted Ca^2+^-triggered spontaneous release of IHCs and reduced vesicle replenishment.

Previous studies showed that otoferlin abundance at the IHC plasma membrane correlates with the presynaptic function and sound encoding (Strenzke et al., 2016). *Otof^Pga/Pga^* mice, wherein otoferlin membrane abundance is attenuated down to 3% of *Otof ^+/+^* mice (Pangršič et al., 2010; Strenzke et al., 2016), displays lower EPSC frequency upon high K^+^-induced IHC depolarization. Although our combination of postsynaptic recordings and high K^+^ stimulation cannot track fast stimulus-secretion coupling, the robust EPSC rates in *Otof^Pga/Pga^* afferents (Figure 1A) suggests that Ca^2+^-triggered membrane fusion is intact despite the mutation in the C_2_F domain (i.e., D1767G, Pangršič et al., 2010). On the other hand, the dual mutations in the C_2_C domain (i.e., D515A and D517A), which alter its Ca^2+^-binding (Johnson and Chapman, 2010) but preserves otoferlin level (Michalski et al., 2017), impairs both vesicle fusion and replenishment functions of otoferlin, suggesting that Ca^2+^-sensing of otoferlin is critical for both steps of exocytosis (Michalski et al., 2017). Thus, not only Ca^2+^-sensing capacity but also plasma membrane level of otoferlin is essential for IHC exocytosis.

### Multiquantal Versus Uniquantal Release: Can Otoferlin Disruption Provide Insight?

The heterogeneity of EPSC amplitude and shape is a hallmark of ribbon synapses, but the underlying mechanisms are not well understood. Notably, the mammalian IHC ribbon synapse differs from other ribbon synapses such as the amphibian and reptile hair cell synapses (Li et al., 2009; Graydon et al., 2011; Schnee et al., 2013) as well as the mammalian retinal bipolar-AII cell synapse (Singer et al., 2004; Mehta et al., 2013), where low [Ca^2+^]_i_ conditions break EPSCs down into unitary events. In contrast, at the rodent IHC ribbon synapse the EPSC amplitude remains sizable despite the massive buffering of presynaptic Ca^2+^ (Goutman and Glowatzki, 2007) or abolition of Ca^2+^ influx (Chapochnikov et al., 2014). This and other findings have led us to consider an alternative hypothesis for explaining EPSC amplitude and shape heterogeneity at the IHC synapse: the large EPSC amplitude may reflect activation of a large number of postsynaptic AMPA receptors (Saito, 1990; Meyer et al., 2009) activated by glutamate liberated from a single synaptic vesicle. Post-fusion regulation of such uniquantal release by a dynamic fusion pore may explain multiphasic EPSCs (successive bouts of release through a flickering pore) and small monophasic EPSCs (incomplete release of a vesicle’s glutamate content) (Chapochnikov et al., 2014; Huang and Moser, 2018; for review, see Moser and Vogl, 2016; Takago and Oshima-Takago, 2018).

Given the presence of large monophasic EPSCs at the *Otof^-/-^* IHC afferent synapse (Figure 2), i.e., under conditions of strongly reduced rate of release that seem not permissive for synchronized fusion of multiple vesicles, we favor the interpretation that uniquantal release prevails in IHCs. Interestingly, the fraction of multiphasic EPSCs was smaller when otoferlin was disrupted. Within the framework of the uniquantal release hypothesis of IHC exocytosis this can be explained as favoring full-collapse fusion and/or singular fusion pore openings. Accordingly, otoferlin would then enhance promote fusion pore flickering. Alternatively, within the framework of multiquantal release hypothesis, which the present study cannot exclude, our observation might be explained as highly synchronized fusion of a smaller number of vesicles upon *Otof* disruption. Besides, either hypothetic mechanism might be modulated by altered Ca^2+^ signaling at the release site as might have resulted from the scaffolding function of Otoferlin for Ca^2+^ channels (Hams et al., 2017) or attenuated fast-inactivating Ca^2+^ currents in Otoferlin-lacking IHCs (Vincent et al., 2017). However, we note that we had previously found normal Ca^2+^ signals at the individual IHC AZs in *Otof^Pga/Pga^* mice (Pangršič et al., 2010). Further testing these hypotheses and elucidating the underlying molecular events remain important goals for future studies.

A smaller fraction of multiphasic EPSCs was also found in *Otof^Pga/Pga^* SGNs (Figure 5) and we suggest that the reduction of otoferlin levels at the plasma membrane suffices to alter the IHC exocytic mode. Tens of missense mutations have been described for patients with otoferlin-related deafness (for review, see Pangršič et al., 2012; Santarelli et al., 2015). It is noteworthy that even a missense mutation of otoferlin such as D1767G (pachanga) drastically decrease the heterogeneity of EPSC shape (this study). Although the functional significance of multiphasic EPSCs at the IHC ribbon synapse remains to be clarified, a wide range of spike jitter in the SGN afferent boutons caused by variable EPSP waveforms (Rutherford et al., 2012) may contribute to the heterogeneity of auditory nerve fiber responses in response to sound (Liberman, 1982; Grant et al., 2010). By promoting the shift in IHC exocytic mode, otoferlin may serve to endow the diversity in sound encoding at this ribbon-type synapse.

## Author Contributions

HT and TM designed the study. HT and TO-T performed experiments and analyzed the data. HT, TO-T, and TM wrote the manuscript. The experiments were performed at the University Medical Center Göttingen and Research Institute of National Rehabilitation Center for Persons with Disabilities.

## Conflict of Interest Statement

The authors declare that the research was conducted in the absence of any commercial or financial relationships that could be construed as a potential conflict of interest.

## Supplementary Material

Figure S1 High K^+^ stimulation does not alter the EPSC size distribution in wild-type or Otoferlin mutant SGNs **(A–C)** Cummulative histograms of monophasic EPSC amplitude **(A)**, multiphasic EPSC amplitude **(B)** and EPSC charge transfer **(C)** derived from exemplar *Otof ^+/+^*, *Otof^Pga/Pga^* and *Otof ^-/-^* SGNs before (5.8 mM [K^+^]_e_, dotted lines) and during high K^+^ (40 mM [K^+^]_e_, solid lines) stimulation. No significant differences in each phenotype.

Figure S2 High K^+^ stimulation does not alter the EPSC kinetics distribution in wild-type or Otoferlin mutant SGNs **(A,B)** Cummulative histograms of 10–90 % rise time **(A1)** and dacay time constant **(A2)** of monophasic EPSCs as well as time to rise **(B1)** and half-width **(B2)** of multiphasic EPSCs derived from exemplar *Otof ^+/+^*, *Otof^Pga/Pga^* and *Otof ^-/-^* SGNs before (5.8 mM [K^+^]_e_, dotted lines) and during high K^+^ (40 mM [K^+^]_e_, solid lines) stimulation. No significant differences in each phenotype.
