# Supplementary figures and images for: Disruption of Otoferlin Alters the Mode of Exocytosis at the Mouse Inner Hair Cell Ribbon Synapse
Source: Front Mol Neurosci. 2019 Jan 9;11:492. doi: 10.3389/fnmol.2018.00492 (PMC6338019; doi:10.3389/fnmol.2018.00492)

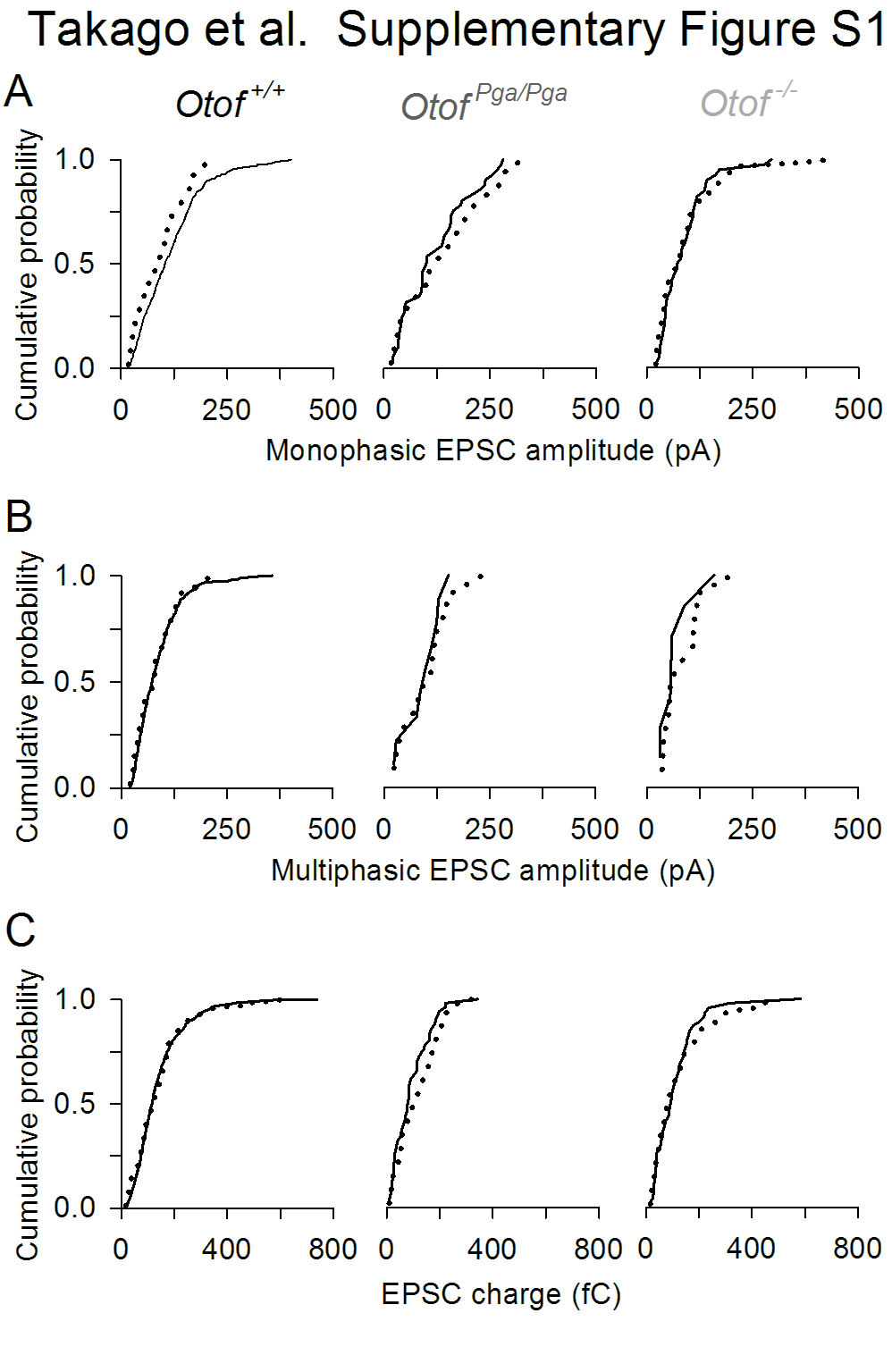

Supplement: Figure S1 — High K+ stimulation does not alter the EPSC size distribution in wild-type or Otoferlin mutant SGNs (A–C) Cummulative histograms of monophasic EPSC amplitude (A), multiphasic EPSC amplitude (B) and EPSC charge transfer (C) derived from exemplar Otof +/+, OtofPga/Pga and Otof -/- SGNs before (5.8 mM [K+]e, dotted lines) and during high K+ (40 mM [K+]e, solid lines) stimulation. No significant differences in each phenotype. [file Image_1.TIF]

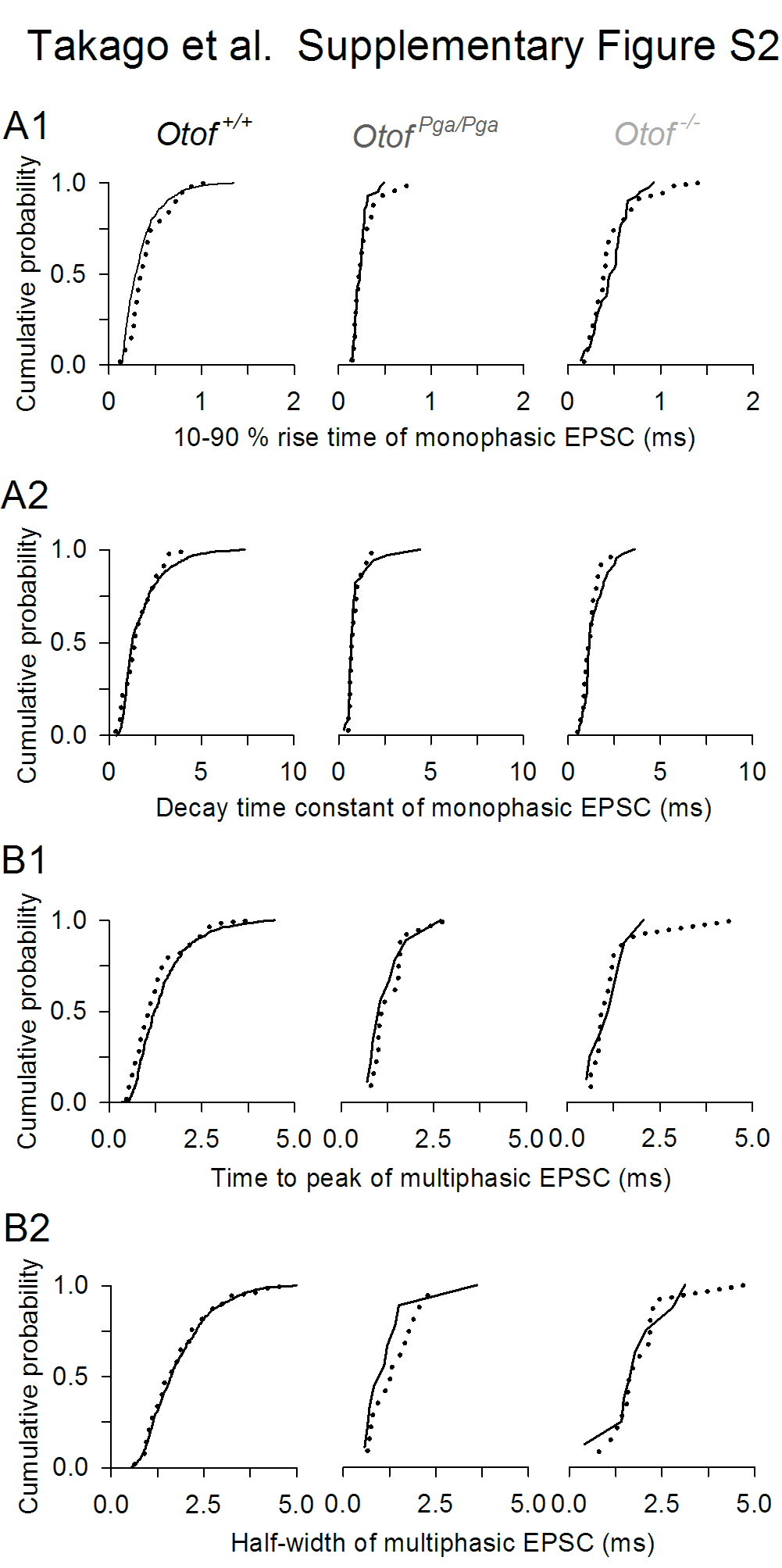

Supplement: Figure S2 — High K+ stimulation does not alter the EPSC kinetics distribution in wild-type or Otoferlin mutant SGNs (A,B) Cummulative histograms of 10–90 % rise time (A1) and dacay time constant (A2) of monophasic EPSCs as well as time to rise (B1) and half-width (B2) of multiphasic EPSCs derived from exemplar Otof +/+, OtofPga/Pga and Otof -/- SGNs before (5.8 mM [K+]e, dotted lines) and during high K+ (40 mM [K+]e, solid lines) stimulation. No significant differences in each phenotype. [file Image_2.TIF]
